# A case of Phaeohyphomycosis caused by *Corynespora cassiicola* infection

**DOI:** 10.1186/s12879-018-3342-z

**Published:** 2018-08-31

**Authors:** Zhaolu Xie, Wei Wu, Desheng Meng, Qing Zhang, Yunqi Ma, Wen Liu, Jianhong Chen

**Affiliations:** Department of Pharmacy, Daping Hospital & Research Institute of Surgery, Army Medical University, Chongqing, 400042 China

**Keywords:** Phaeohyphomycosis, Corynespora cassiicola, Voriconazole

## Abstract

**Background:**

*Corynespora cassiicola* infection is common in plants, but the human *Corynespora cassiicola* infection in our report is rare according to the literature.

**Case presentation:**

We report a case of subcutaneous phaeohyphomycosis caused by a plant pathogen in a patient with acute heart failure. The organism was isolated and identified as *Corynespora cassiicola* according to its morphological characteristics and gene analysis. The patient was treated successfully with systemic voriconazole.

**Conclusions:**

This is the third reported case of subcutaneous infection caused by *Corynespora cassiicola* and the first reported case with accompanied renal impairment, which was associated with acute heart failure. Our case also suggests the importance of renal function monitoring in patients receiving intravenous voriconazole treatment.

**Electronic supplementary material:**

The online version of this article (10.1186/s12879-018-3342-z) contains supplementary material, which is available to authorized users.

## Introduction

*Corynespora cassiicola* is a fungal phytopathogen found in many plant species [1]. It has been found to infect diverse parts of plants such as leaves, stems, and roots [2]. Although it has been defined as plant pathogen, it opportunistically infects humans and rarely causes phaeohyphomycosis. Phaeohyphomycosis indicates the infections caused by dematiaceous fungi, including superficial, cutaneous and subcutaneous infections. The infection may involve the central nervous system as well [3]. Here we report a case of subcutaneous phaeohyphomycosis caused by *Corynespora cassiicola*. The renal function of the patient was impaired, which is a crucial factor for the effective and safe selection among antifungal drugs.

## Case presentation

**Fig. 1:**
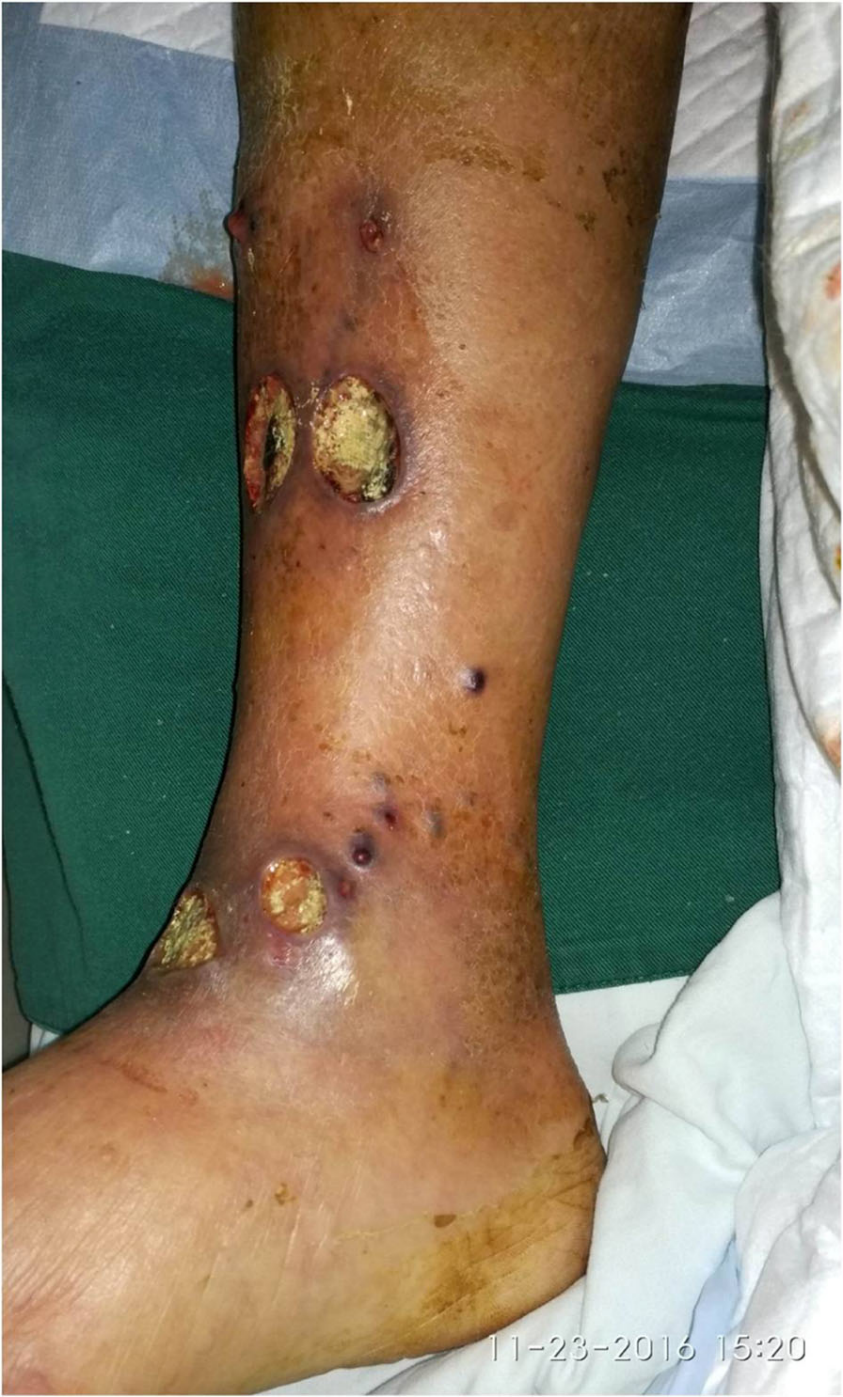
Ulcerative lesions on the right leg upon patient admission

A 76-year-old male was referred to the hospital because of acute heart failure, AECOPD and ulcers on the right leg. The ulcers were scattered as multifocal lesions with the size of 2–4 cm, with purulent discharge (Fig. 1). The ulcers developed in two months and the patient denied histories of trauma and plant cultivation. There was 1-year history of COPD, 11-year history of hypertension, and no history of diabetes mellitus (Additional file 1).

On admission, the patient was dyspneic with a respiratory rate of 30 breaths per minute. Physical examination revealed wheezing and moist rale in both lungs. Slight pitting edema and cyanosis were observed at cold extremities. Results of routine laboratory tests were as follows: WBC 18 × 10^9^/mL; N% 92; CRP 15 mg/L; PCT 0.74 ng/mL; and GFR (glomerular filtration rate) 32 mL/min. Arterial blood gas analysis showed an acute decompensated metabolic acidosis. Several Gram stains from the ulcers showed moderate amount of Gram-negative bacteria and large amount of leukocytes. Chest X-ray showed pulmonary infection (Additional file 2: Figure S1A). The patient received treatment for heart failure, and incision and drainage of purulent spots. Anti-infection therapy was initiated with cefoperazone/sulbactam (2:1).

Three days after the treatment, the dyspnea was apparently remitted and wheezing and moist rale in the lung disappeared. The microbiological culture of the pus obtained on the day of admission revealed colonies of *Klebsiella pneumonia* and *Proteus vulgaris*. Laboratory reexamination showed elevated PCT (3.86 ng/mL), N% (90%) and CRP (66 mg/L). BDG (1, 3-β-D-glucan) was 554 pg/mL. Echocardiography displayed no vegetation. Pulmonary infection was improved according to the chest X-ray (Additional file 2: Figure S1B). Antibiotic treatment was terminated and adequate dressing change was assured.

**Fig. 2:**
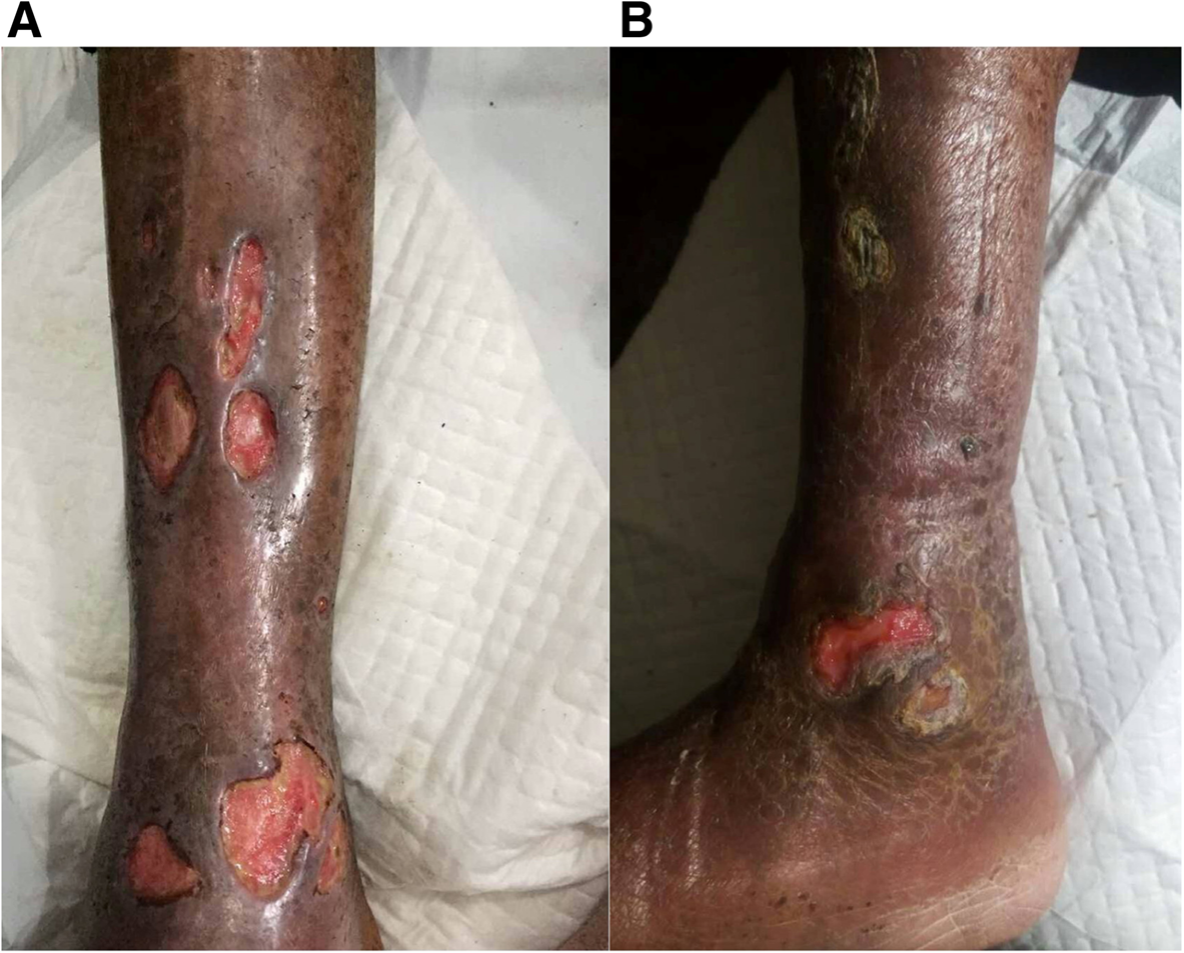
Alleviated lesions after anti-infection therapy. **a**) One week after voriconazole treatment, the surface of the lesions became dry and cleaner. **b**) Six weeks after voriconazole treatment, lesions crusted and showed signs of healing

**Fig. 3:**
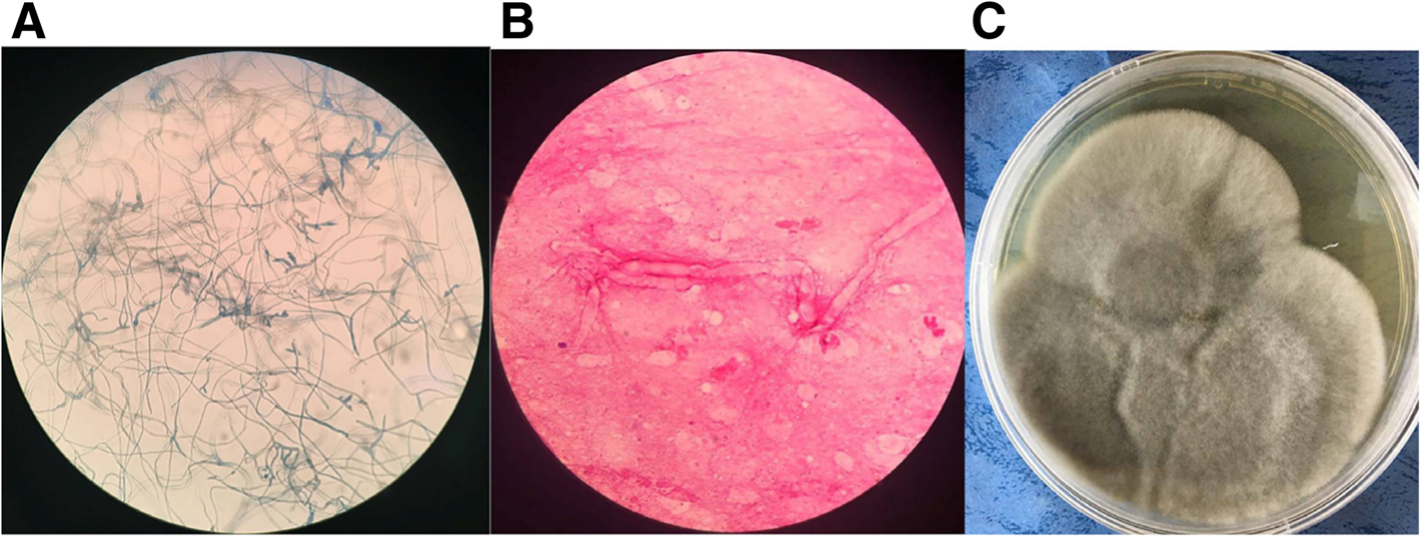
Morphology of the isolate. **a**) Micrograph of the isolate stained with Lactophenol cotton blue. **b**) Microscopy after Gram stain. **c**) Colonies incubated on Sabouraud dextrose agar at 25 °C

On the seventh day of hospitalization, the patient did not show dyspnea, but the body temperature rose to 38.5 °C. The surface of ulcers on the right leg was dry and granulation was observed but with no evidence for further wound healing. Laboratory reexamination showed abnormal PCT (1.02 ng/mL), N% (85%) and CRP (68 mg/L). GFR increased to 51 mL/min. Pus obtained from deep part of the ulcer was tested and fungal hyphae were found in smear examination. MRI of the right leg indicated the skin damage as inflammatory changes. Voriconazole and piperacillin/tazobactam were administrated intravenously for suspected infection. The patient did not show fever again. Three days after intravenous administration of voriconazole, GFR dropped to 40 mL/min. Intravenous voriconazole treatment was then terminated and replaced by oral voriconazole. The lesions were obviously attenuated with anti-infection treatment and adequate dressing change (Fig. 2a). The fungal culture of the pus obtained after admission revealed filamentous fungi for several times. The isolated fungi were further identified as *Corynespora cassiicola* by morphological characteristics and gene analysis (Fig. 3). Laboratory tests showed improvement in PCT (0.9 ng/mL), N% (67%) and CRP (40 mg/L).

Two weeks after voriconazole treatment, the patient was discharged but oral voriconazole treatment continued at the out-patient clinic. A month after the discharge, most of the lesions crusted and showed signs of healing (Fig. 2b). The voriconazole treatment lasted for 6 weeks was terminated and the lesions were washed with potassium permanganate (1:5000) once every day. At the 2-month follow-up, all lesions healed completely.

## Discussion and conclusions

Phaeohyphomycosis is a heterogeneous group of infections induced in human by opportunistic dematiaceous fungi. More than 50 genus and 100 species of pathogenic fungi are able to cause phaeohyphomycosis. Most of the pathogenic fungi are saprotropic fungi living in the soil or plants, but they may invade human with traumatic origin. Their infections in human could be in skin, cornea, and subcutaneous tissues and even be systemic [4]. However, phaeohyphomycoses are usually unrecognized or misidentified because the pathogens lack morphological specificity and present polymorphic appearance.

*Corynespora cassiicola* is a common plant pathogen, which has a broad host range worldwide. It can infect many plants in tropical and subtropical climate zones, including cucumber, tomato, rubber plant and cotton [5]. Human infections by *Corynespora cassiicola* are rare and thus far only four cases have been reported in the literature. The first case was reported in 1969 [6], in which *Corynespora cassiicola* was isolated from a patient with Madura foot in the Sudan. Huang and his colleague described a farmer with subcutaneous infection in both hands caused by *Corynespora cassiicola* [7]. Another subcutaneous infection caused by *Corynespora cassiicola* was also reported afterwards [4]. Moreover, *Corynespora cassiicola* has also been isolated and identified in a corneal infection [8].

The management of phaeohyphomycosis with itraconazole or voriconazole is recommended strongly but only based on lower level evidence [9]. For the treatment of *Corynespora cassiicola* infection, terbinafine [4] and amphotericin B [7] have also shown success. More specifically, the patient with corneal infection healed after receiving the treatment with topical voriconazole, micafungin and pimaricin for 3 months and intravenous voriconazole for 1 month [8]. Subcutaneous infections in both hands were treated successfully with amphotericin B after ineffective oral itraconazole therapy [7]. And subcutaneous infections in the legs were cured with oral terbinafine and topical povidone iodine for 6 weeks [4].

The patient in the current report was referred to our hospital mainly because of acute heart failure rather than the ulcer on the right leg. According to the description of the patient, it had been nearly two months since the ulcer occurred, which was not taken seriously. *Corynespora cassiicola* infection is generally found in immunocompromised patients. It is worth to note that the patient in current report is 76 years old with COPD and poor nutritional status, which may contribute to the right leg infection.

As the several Gram stains from the ulcer showed Gram-negative bacteria and large amount of leukocytes, cefoperazone/sulbactam was applied as an empiric antibacterial therapy. Meanwhile, the use of cefoperazone/sulbactam also aims at the control of pulmonary infection because cefoperazone/sulbactam is effective for the treatment of lower respiratory tract infections as well as skin and soft tissue infections. While the antibacterial therapy was successful for pulmonary infection, no obvious relief was observed on the ulcer on the right leg. Considering the unsatisfactory result might be associated with the pathogens out of the cefoperazone/sulbactam antibacterial spectrum, proofs for fungal infection had been investigated and antifungal therapy was employed afterwards.

Although the pathogenic fungus was not identified until the discharge, fortunately the voriconazole treatment was successful. Voriconazole was chosen for initial antifungal therapy based on several factors. Firstly, the antifungal spectrum of voriconazole is important. The smear examination and fungal culture revealed filamentous fungi, and voriconazole is the first-line therapy of most filamentous fungi according to the guideline [10]. Secondly, the pharmacokinetic features should be taken into account. Voriconazole is supposed to be well distributed in the skin and soft tissue, and it is a weaker inhibitor of P450 enzymes among all the azole antifungals, suggesting less potential drug interactions. Coincidently, the patient was receiving the treatment with multiple drugs but no specific drug interaction was found. Another fact need to be paid attention to is that sulfobutylether-betacyclodextrin (SBECD) was used as the solvent for intravenous administration of voriconazole. Lipophilic voriconazole was contained in the center of SBECD to enhance its solubility and stability [11, 12]. Ninety five percent of SBECD are renally excreted, and SBECD will accumulate in patients with moderate to severe renal impairment [13]. At the meantime, oral bioavailability of voriconazole is up to 96%. Consequently, oral instead of intravenous voriconazole is recommended when the GFR is < 50 mL/min [10, 14]. In this case, renal impairment occurred after acute heart failure. GFR of the patient was 51 mL/min when voriconazole treatment started. Based on renal function test, oral voriconazole had been timely started as the substitute to avoiding further renal impairment.

In summary, our patient was referred to the hospital due to acute heart failure and fortunately took the chance to deal with the ulcer on his right leg. During hospitalization, infection on right leg gradually became the major problem. *Corynespora cassiicola* was finally identified as the causative pathogen, which is rare to be seen in humans. This case also reminds us that monitoring of renal function and proper transition to oral formulation will be crucial for patients with reduced GFR when receiving intravenous administration of voriconazole.

## Additional files

Additional file 1: Timeline of the case. Patient clinical course. (DOCX 39 kb)

Additional file 2: Supplementary figure. Chest X-ray. (DOCX 2295 kb)

## Abbreviations

AECOPD: Acute exacerbation of chronic obstructive pulmonary disease
BDG: 1, 3-β-D-glucan
COPD: Chronic obstructive pulmonary disease
CRP: C-reactive protein
GFR: Glomerular filtration rate
PCT: Procalcitonin
SBECD: Sulfobutylether-betacyclodextrin
WBC: White blood cell
