# Supplementary material for: A case of Phaeohyphomycosis caused by Corynespora cassiicola infection
Source: BMC Infect Dis. 2018 Aug 31;18:444. doi: 10.1186/s12879-018-3342-z (PMC6119301; doi:10.1186/s12879-018-3342-z)
Supplement: Supplementary file 1 — Timeline of the case. Patient clinical course. (DOCX 39 kb) [file 12879_2018_3342_MOESM1_ESM.docx]

76-year-old male, 1-year history of COPD, no history of diabetes

**Day 21**

**Day 10**

Gene analysis of the isolate (after discharge): *Corynespora cassiicola*

Lesions obviously attenuated

Laboratory tests: lower infection markers

Pus culture: filamentous fungi

Morphology of the isolate: dematiaceous fungi with septahypha

Laboratory tests: GFR dropped to 40 mL/min

No dyspnea, temperature 38.5 ℃. The surface of ulcers was dry and granulation was observed but with no evidence for further wound healing.

Laboratory tests: lower infection markers, higher GFR

MRI of the leg: skin damage as inflammatory changes

Pus smear exam: fungal hyphae

Intravenous voriconazole treatment was then terminated and replaced by oral voriconazole

Not show fever again

Dyspnea apparently remitted

Physical exam: wheezing and moist rale in the lung disappeared

Laboratory tests: higher infection markers, BDG positive

Chest X-ray: pulmonary infection improved

Pus culture: *Klebsiella pneumonia* and *Proteus vulgaris*

Treatment for heart failure, and incision and drainage of purulent spots

Anti-infection therapy initiated with cefoperazone/sulbactam

The patient was dyspneic and the ulcers were scattered as multifocal lesions with purulent discharge.

Physical exam: wheezing and moist rale in both lungs

Laboratory tests: increased infection markers, decreased GFR

Chest X-ray: pulmonary infection

Grams stain from the ulcers: Gram-negative bacteria and large amount of leukocytes

Discharged from hospital with oral voriconazole

Lesions healed completely on following up at 8 wks

Antibiotic treatment was terminated and adequate dressing change was assured

Voriconazole and piperacillin/tazobactam were administrated intravenously

**Day 4**

**Day 1**

**Day 7**
