# Supplementary figures and images for: A case of Phaeohyphomycosis caused by Corynespora cassiicola infection
Source: BMC Infect Dis. 2018 Aug 31;18:444. doi: 10.1186/s12879-018-3342-z (PMC6119301; doi:10.1186/s12879-018-3342-z)

**Supplementary figure**


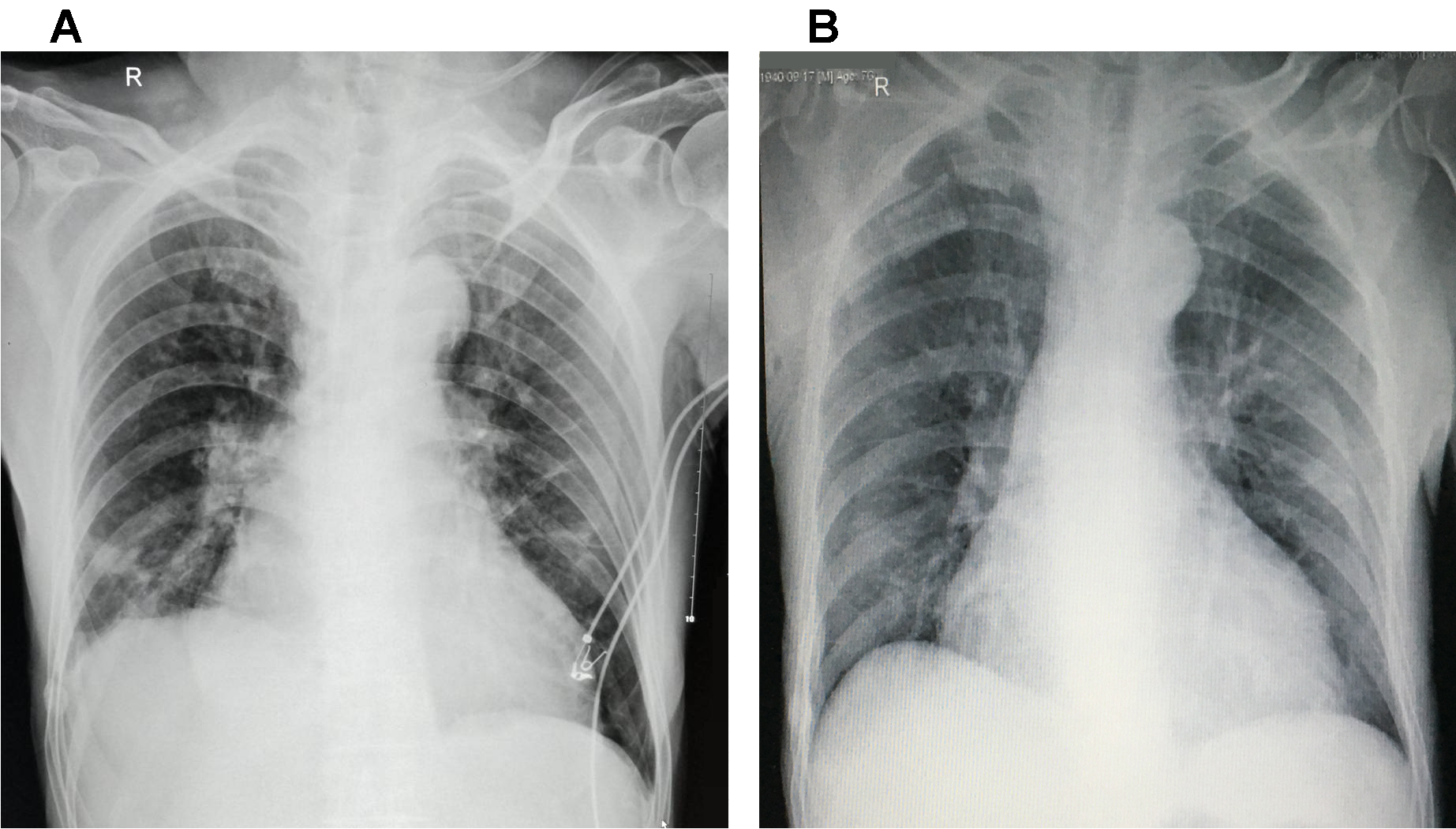


**Fig. S1 Chest X-ray before A) and after B) antibacterial treatment.**

Supplement: Supplementary file 2 — Supplementary figure. Chest X-ray. (DOCX 2295 kb) [file 12879_2018_3342_MOESM2_ESM.docx]
